# Draft genome sequences for three unisolated *Alnus*-infective *Frankia* Sp+ strains, AgTrS, AiOr and AvVan, the first sequenced *Frankia* strains able to sporulate *in-planta*

**DOI:** 10.7150/jgen.35875

**Published:** 2019-09-17

**Authors:** Lorine Bethencourt, Florian Vautrin, Najwa Taib, Audrey Dubost, Lucia Castro-Garcia, Olivier Imbaud, Danis Abrouk, Pascale Fournier, Jérôme Briolay, Agnès Nguyen, Philippe Normand, Maria P. Fernandez, Céline Brochier-Armanet, Aude Herrera-Belaroussi

**Affiliations:** 1 Univ Lyon, Université Lyon 1, CNRS, UMR5557, Ecologie Microbienne, INRA, UMR 1418, 43 bd du 11 novembre 1918, F-69622 Villeurbanne, France; 2 Univ Lyon, Université Lyon 1, CNRS, UMR5558, Laboratoire de Biométrie et Biologie Évolutive, 43 bd du 11 novembre 1918, F-69622 Villeurbanne, France; 3 Univ Lyon, Université Lyon 1, DTAMB, FR 3728 BioEnviS, 43 bd du 11 novembre 1918, F-69622 Villeurbanne, France; 4 Biofidal, 170 av Gabriel Péri, F-69518 Vaulx-en-Velin, France

**Keywords:** *Frankia*, AgTrS, AiOr, AvVan

## Abstract

Actinobacteria from genus* Frankia* are able to form symbiotic associations with actinorhizal plants including alders. Among them, Sp+ strains are characterized by their ability to differentiate numerous sporangia inside host plant cells (unlike “Sp-” strains unable of *in-planta* sporulation). Here, we report the first genome sequences of three unisolated Sp+ strains: AgTrS, AiOr and AvVan obtained from *Alnus glutinosa*, *A. incana* and *A. alnobetula* (previously known as *viridis*), respectively (with genome completeness estimated at more than 98%). They represent new *Frankia* species based on Average Nucleotide Identity (ANI) calculations, and the smallest *Alnus*-infective *Frankia* genomes so far sequenced (~5 Mbp), with 5,178, 6,192 and 5,751 candidate protein-encoding genes for AgTrS, AiOr and AvVan, respectively.

## Genome Announcement

*Frankia* strains are filamentous actinobacteria able to fix nitrogen and to form symbiotic associations with actinorhizal plants, leading to the formation of root nodules where trophic exchanges between plant and bacteria take place. Phylogenetic studies showed that clades within *Frankia* genus are strongly linked to infection groups, with Cluster 1 containing strains infective on *Alnus* and *Myrica* 1, 2. *Frankia* is also characterized by its ability to differentiate sporangia. Most isolated *Frankia* strains have been described as sporulating *in-vitro* 1, 3. However, certain strains, called “Sp+”, have the ability to sporulate inside host cells (unlike “Sp-” strains unable of *in-planta* sporulation) 4. Sp+ strains have been commonly reported in association with alders, especially *A. glutinosa*, *A. incana* and *A. alnobetula* (formerly *A. viridis*) species. In contrast to Sp- strains, up to date, Sp+ strains are still totally culture recalcitrant (none are available in pure culture despite many isolation attempts) 5. Furthermore, we recently described their narrower host specificity 6, suggesting a strong host dependence. It was hypothesized that Sp+ strains could have evolved towards an obligatory symbiont status with spores representing their only form of survival outside the host. Indeed, produced early and abundantly in host cells, spores would be released during nodule senescence, thus enabling Sp+ strains to survive and disseminate in the soil in a free state 7. Recently, MLSA-based studies directly conducted on Sp+ nodules collected from various geographical sites confirmed that *Alnus*-infective Sp+ strains belonged to Cluster 1 as expected. These studies also showed that the Sp+ trait was associated with distinct phylogenetic lineages, strongly correlated to the host species 7, 2, suggesting that Sp+ strains had emerged several times independently over the course of *Frankia* diversification. To date, more than thirty *Frankia* strains covering the diversity of the *Frankia* genus have been sequenced 8, helping to predict and identify pathways involved in the biosynthesis of natural products by *Frankia* 9, 10. However, no *Frankia* Sp+ genomes have been reported so far. Here, we reported the sequencing of three Sp+ *Frankia* genomes. The main challenge was to get DNA of these unisolated strains directly from nodules, limiting plant DNA contaminations. For this, we optimized a protocol of DNA extraction from *Frankia* spores directly isolated from nodules.

Three *Alnus*-infective Sp+ *Frankia* uncultured strains from Cluster 1, AgTrS, AiOr and AvVan, were selected from nodules collected in 3 distinct alder stands, colonized by *A. glutinosa* (Le Tremblay, Savoie, France), *A. incana* (Ornon, Isère, France) and *A. alnobetula* (Vanoise, Savoie, France), respectively 6. AgTrS, AiOr and AvVan genomes were sequenced using DNA extracted from spores directly isolated from nodules. For each strain, at least 1 g of surface-sterilized (with calcium hypochlorite 1 % w/v, 15 minutes) and peeled nodules was crushed in liquid nitrogen, with 10 mL of buffer containing 0.5 M Tris-HCl pH 7, 4 % PVP (w/v), 0.1 M KCl, 5 mM EDTA, 0.6 M sucrose, 10 mM Na_2_S_2_O_3_. Crushed nodule suspensions were successively filtered through 100 µM and 20 µM sterile-filters (Steriflip® Filter Millipore, Life Science-Merck, Paris, France) to separate *Frankia* spores from plant residues and *Frankia* hyphae and vesicules. *Frankia* spore suspensions were homogenized using TissueLyser II, (Qiagen, Courtaboeuf, France) for 40 sec. at 20 Hz before total DNA extraction. DNA was extracted with FastDNA® SPIN for Soil kit (MP Biomedicals, Illkirch, France) following the supplied procedure. DNA samples were shotgun sequenced after a Nextera XT library construction step (Illumina, USA), using Illumina MiSeq technology with a paired-end 2 × 300-bp run (MiSeq 600 cycles V3 kit, Biofidal, Lyon, France). It is worth noting that an additional sequencing was conducted on AgTrS strain using 454-pyrosequencing technology (Life Sciences- Merck), however this did not allow to improve genome assembly and was thus not performed for the two other strains AiOr and AvVan. Genome assemblies were realised using Unicycler v0.4.3 11 and their annotation was done with the MicroScope platform version 3.10.0 12.

A total of 3,480,805 reads were generated for AgTrs, 4,413,305 reads for AiOr and 1,805,928 reads for AvVan. Reads were sorted by nucleotide frequencies using Perl scripts to remove the reads with G+C content ≤ 54 %, since they are likely due to host plant DNA contaminations. More precisely, this threshold was based on the high G+C content reported in *Frankia* genomes 13, with a 72% overall G+C content (only 26 short genes below 54% GC and a single group of 5 very short genes below 54% G+C), against a mean G+C content of alder genomes of ~40% 14. Based on G+C content read sorting, a final set of 2,401,363 reads was retained for AgTrS, 3,977,168 reads for AiOr and 549,771 reads for AvVan. Seventy-six to 96% of eliminated reads from AgTrS, AiOr and AvVan sequencing data showed percent sequence identity ID > 85 % against *A. glutinosa* genome (accession no. ASM325496v1) and less than 1% against *Frankia* genomes on MicroScope platform (only 0.1 and 0.2% for AgTrS and AvVan, respectively). Genome assemblies based on sorted reads showed a reduced number of contigs as well as an increased mean contig size compared to assemblies based on unsorted reads, suggesting a significant improvement of genome assemblies (Table 1).

**Figure 1:**
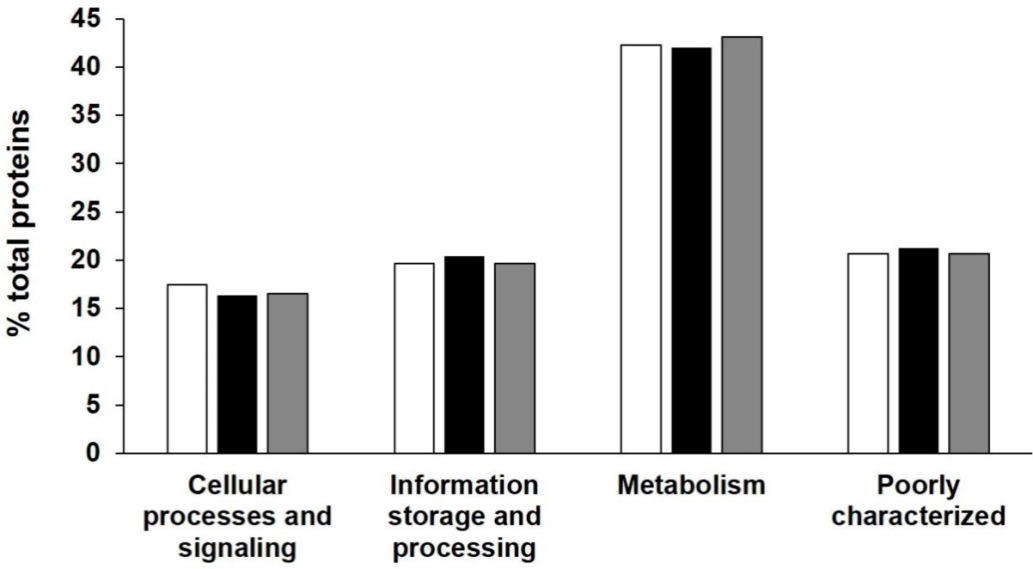
** COG functional classification of proteins encoded on the three sequenced Sp+ *Frankia* genomes AgTrS, AiOr and AvVan.** Proportions (%) of proteins in each of the COG super-functional categories “Cellular processes and signalling”, “Information processing and storage”, “Metabolism” and “Poorly characterized”, predicted for AgTrS (white bars), AiOr (black bars) and AvVan (grey bars) genomes.

Assembly data are summarized in Table 2 together with genomes associated with *Frankia* species, already described or soon to be. The final draft assembly for AgTrS consisted of 281 contigs (≥ 5 kb). The maximum length and N50 values of the contigs were 96.9 kb and 15.3 kb, respectively, giving a total genome size of 4,882,652 bp. For AiOr, the final draft assembly consisted of 302 contigs (≥ 5 kp) containing 5,504,816 bp, with a maximum contig length of 105.2 kb and a N50 value of 17.4 kb. Both AgTrS and AiOr draft genomes had an overall G+C content of 71.6%. For AvVan, the final draft assembly consisted of 322 contigs (≥ 5 kb), with the contig maximum length and N50 values of 30.1 kb and 6.6 kb, respectively. It contained a total sequence of 4,877,887 bp, with an overall G+C content of 71.4%. Genome completeness was estimated at 98.1% for AgTrS and AvVan strains and 99.4% for AiOr, using CheckM software that assesses the presence of a specific number of markers depending on the studied organism (307 markers for *Frankia* genomes) 15. The assembled genomes of AgTrS, AiOr and AvVan strains resulted in 5,178, 6,192 and 5,751 candidate protein-encoding genes, respectively (Table 2). Classification of proteins into their COG functional categories (using MicroScope Platform from Genoscope, http://www.genoscope.cns.fr/agc/microscope/home/index.php) showed similar proportions of proteins in the different functional groups among the three strains (Figure 1).

**Figure 2:**
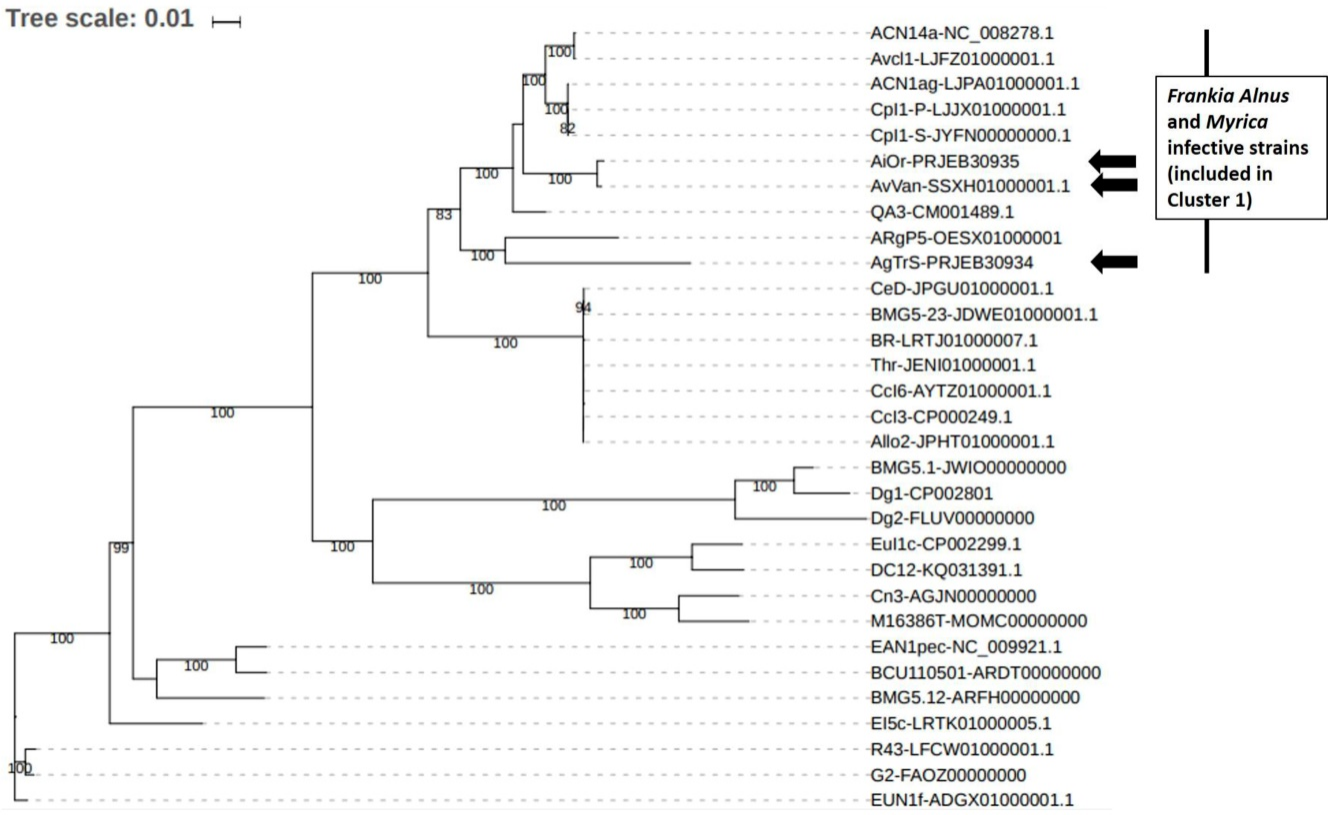
** Position of the three Sp+ sequenced *Frankia* strains AgTrS, AiOr and AvVan in *Frankia* genus phylogeny based on ribosomal proteins.** In addition to Sp+ genomes, a total of 28 sequenced *Frankia* strains were used. For all the 31 genomes, 51 ribosomal protein sequences (total size = 18,582 nt) were included in a supermatrix and the phylogenetic tree was constructed based on the model GTR+I+R4.

*Frankia* sp. AgTrS, AiOr and AvVan Sp+ strains represent the smallest *Alnus*-infective *Frankia* genomes so far sequenced (~5 Mbp), close to the genome size of *Casuarina*-infective strains previously described as subservient to their host 16. In order to place the three Sp+ strains in *Frankia* reference phylogeny and to assess the relationships between them, a maximum likelihood phylogeny was inferred (Figure 2). More precisely, the 28 *Frankia* genomes available from NCBI were retrieved (for all these strains, origins and genome features have been summarized by Tisa et al. 8) and gathered in a local database together with the 3 Sp+ assemblies. This dataset included seven strains from Cluster 1 unable to sporulate *in-planta*, thus Sp- strains: ACN14a as *F. alni* species representative, AvcI1, ACN1ag, CpI1P, CpI1S, QA3 and ARgP5. Fifty-one ribosomal proteins were retrieved from the 31 genomes and combined to build a large supermatrix of (18,582 nucleotide positions) that was used for phylogenetic inferences. The ML tree was built with IQ TREE 17 with the GTR+I+R4 evolutionary model as suggested by the model selection tool implemented in IQ TREE. The branch robustness of the ML tree was estimated with the non-parametric bootstrap procedure Implemented in IQ TREE (100 replicates of the original alignment). The resulting tree confirmed the position of the 3 Sp+ *Frankia* strains AgTrS, AiOr and AvVan into Cluster 1 (Figure 2). In this cluster, AvVan and AiOr appeared closely related to ACN14a, AvcI1, ACN1ag, Cpl1P, Cpl1S, and QA3 strains (bootstrap value = 100%), while AgTrS formed a distinct lineage (Figure 2), suggesting that the three Sp+ strains belonged to two different clades as previously discussed 2, 7.

Average Nucleotide Identity (ANI) calculations were performed in order to accurately distinguish between strains at the species level into the Cluster 1, using the recommended cut-off point of 95 % ANI for species delineation 18. All 3 Sp+ *Frankia* genomes AgTrS, AiOr, and AvVan showed less than 90.1% similarity with the genomes of ACN14a and QA3 *Alnus*-infective *Frankia* strains from Cluster 1 (both ACN14a and QA3 have also been included in the phylogenetic tree in Figure 2). Only two genomes, AvVan and AiOr shared 98.5% ANI, which is above the threshold value for species circumscription. These phylogenomic analyses confirm the results obtained by a large survey on Sp+ strains that showed the genetic divergence between *A. glutinosa*-infective strains and *A. alnobetula*- and *A. incana*-infective strains 2, 7. These results lead to conclude that AgTrS, AiOr and AvVan most likely represent two new distinct species into Cluster 1 of *Frankia* genus, with AiOr and AvVan belonging to the same species.

In conclusion, the genome sequencing of the three *Frankia* Sp+ strains AgTrS, AiOr and AvVan offer a unique opportunity to explore the evolution of their life history traits. Thorough analyses based on comparative genomic approaches with *Frankia* Sp- genomes already available will be performed, for instance to look for clues to Sp+ strain ability to sporulate *in-planta*, to their non-cultivability/host dependence, to their higher narrower host specificity, and eventually clarify their hypothetical status of obligatory symbiont.

## Nucleotide sequence accession numbers

This whole-genome shotgun project has been deposited in DDBJ/EMBL/GenBank under the accession no. PRJEB30934, PRJEB30935 and SSXH00000000 (for *Frankia* sp. AgTrS, AiOr and AvVan). The version described in this paper is the first version. No pure culture of AgTrS, AiOr and AvVan strains are available, these strains are maintained in the UMR5557 Microbial Ecology of Lyon (France) on *Alnus* seedlings (under controlled hydroponic conditions) and they are available as nodules to the research community upon request.

## Figures and Tables

**Table 1:** Assembly data of the three Sp+ *Frankia* genomes sequenced, before and after read sorting based on their G+C content (to remove the reads with G+C content ≤ 54 %).

	Data before read sorting			Data after read sorting		
Sp+ strain	Read number	Total contig number (with ≥ 5 kb)	Mean contig size (pb)	Read number	Total contig number (with ≥ 5 kb)	Mean contig size (pb)
AgTrS	3,480,805	3,559 (366)	2,120	2,401,363 (69%)	612 (281)	7,978
AiOr	4,413 305	1,860 (376)	4,542	3,977,168 (90%)	669 (302)	8,228
AvVan	1,805,928	7,798 (457)	2,876	549,771 (30%)	1,228 (322)	3,962

**Table 2:** Genome features of the three Sp+ *Frankia* genomes sequenced (in grey), compared to available *Frankia* genomes (only genomes associated with described or being described *Frankia* species were included in this table and they are all Sp- strains).

Species	Cluster	Strain	Genome accession #	No. of contigs	Genome length (nt)	Genomic G+C content (mol%)	N50 genomic values	Genome coverage	Genome completeness (%) (CheckM)	Original host genus	Associated hosts	Reference
-	1	AgTrS	PRJEB30934	281 (≥ 5 kb)	4,882,652	71.6	15.3	295.1	98.1	*Alnus*	*A glutinosa, A. incana*	This study, 6
-	1	AiOr	PRJEB30935	302 (≥ 5 kb)	5,504,816	71.6	17.4	433.5	99.4	*Alnus*	*A. incana*	This study, 6
-	1	AvVan	SSXH00000000	322 (≥ 5 kb)	4,877,887	71.4	6.6	67.6	98.1	*Alnus*	*A. viridis*	This study, 6
*F. alni*	1	DSM 45986^T^ (ACN14a^T^)	NC_008278.1	1	7,497,934	72.8	-	-	-	*Alnus*	*Alnus*, Myricaceae	13
*F. torreyi*	1	DSM 44263^T^ (CpI1-S^T^)	JYFN00000000	153	7,624,758	72.4	-	-	-	*Comptonia*	*Alnus*, Myricaceae	19
*F. canadensis*	1	DSM 45898^T^ (ARgP5^T^)	OESX01000001	568	7,730,285	72.4	-	-	-	*Alnus*	*Alnus*, Myricaceae	20
*F. casuarinae*	1	DSM 45818^T^ (CcI3^T^)	CP000249.1	1	5,433,628	70.1	-	-	-	*Casuarina*	Casuarinaceae (except* Gymnostoma*)	13
*F. coriariae*	2	DSM 100624^T^ (BMG5.1^T^)	JWIO00000000	116	5,795,263	71.0	-	-	-	*Coriaria*	*Datisca, Coriaria*	21
*Candidatus F. californiensis*	2	Dg2	FLUV00000000	1066	5,929,312	67.9	-	-	-	*Datisca*	Rosaceae, *Datisca*	22
*Candidatus F. datiscae*	2	Dg1	CP002801	1	5,323,186	70.0	-	-	-	*Datisca*	*Datisca, Coriaria*	23
*F. discariae*	3	DSM 46785^T^ (BCU110501^T^)	ARDT00000000	207	7,891,711	72.3	-	-	-	*Discaria*	Rhamnaceae, Elaeagnaceae, *Gymnostoma*	24
*F. elaeagni*	3	DSM 46783^T^ (BMG5.12^T^)	ARFH00000000	139	7,589,313	71.7	-	-	-	*Elaeagnus*	Rhamnaceae, Elaeagnaceae, *Gymnostoma*	25
*F. irregularis*	3	DSM 45899^T^ (G2^T^)	FAOZ00000000	83	9,537,992	70.9	-	-	-	*Casuarina*	Rhamnaceae, Elaeagnaceae, *Gymnostoma*	26
*F. inefficax*	4	DSM 45817^T^ (EuI1c^T^)	CP002299.1	1	8,815,781	72.3	-	-	-	*Elaeagnus*	Elaeagnaceae, *Morella*	8
*F. saprophytica*	4	DSM 105290^T^ (Cn3^T^)	AGJN00000000	2	9,978,592	71.8	-	-	-	*Coriaria*	-	27
*F. asymbiotica*	4	DSM 100626^T^ (M16386^T^)	MOMC00000000.1	174	9,435,764	72.0	-	-	-	*Morella*	-	28
